# Beyond depression and anxiety; a systematic review about the role of corticotropin-releasing hormone antagonists in diseases of the pelvic and abdominal organs

**DOI:** 10.1371/journal.pone.0264909

**Published:** 2022-03-11

**Authors:** Joshua E. Pagán-Busigó, Jonathan López-Carrasquillo, Caroline B. Appleyard, Annelyn Torres-Reverón

**Affiliations:** 1 Department of Basic Sciences, Ponce Health Sciences University, Ponce Research Institute, Ponce, Puerto Rico, United States of America; 2 Sur180 Therapeutics, LLC, McAllen, Texas, United States of America; Medizinische Universitat Graz, AUSTRIA

## Abstract

Evidence for beneficial effects of corticotropin releasing hormone (CRH) antagonists in abdominal and pelvic organs is emerging in preclinical studies. Following the Preferred Reporting Items for Systematic Reviews and Meta-Analyses (PRISMA) statement a compilation of preclinical studies using CRH receptor antagonists as a treatment for abdominal and pelvic disease was carried out. The Animal Research: Reporting of In Vivo Experiments (ARRIVE) essential 10 guidelines were used to determine quality of the included studies. A total of 40 studies from the last 15 years studying irritable bowel syndrome, inflammatory bowel disease, endometriosis, enteritis, stress impact on gastrointestinal processes and exogenous CRH administration effects were included. Blockage of the CRH receptor 1 was mainly associated with beneficial effects while that of CRH receptor 2 worsened studied effects. However, time of administration, route of administration and the animal model used, all had an impact on the beneficial outcomes. Frequency of drugs administered indicated that astressin-2B, astressin and antalarmin were among the most utilized antagonists. Of concern, studies included were predominantly carried out in male models only, representing a gender discrepancy in preclinical studies compared to the clinical scenario. The ARRIVE score average was 13 with ~60% of the studies failing to randomize or blind the experimental units. Despite the failure to date of the CRH antagonists in moving across the clinical trials pipeline, there is evidence for their beneficial effects beyond mood disorders. Future pre-clinical studies should be tailored towards effectively predicting the clinical scenario, including reduction of bias and randomization.

## Introduction

Corticotropin-releasing hormone (CRH; also known as corticotrophin releasing factor or CRF) is a 41-amino-acid peptide produced by parvocellular neuroendocrine cells of the hypothalamus. CRH plays a crucial role in organizing the hypothalamic-pituitary-adrenal (HPA) axis and coordinating the immune, behavioral, endocrine, and autonomic responses to stress [1]. CRH is produced and acts both within the central nervous system (CNS) and at several peripheral sites [2]. In the CNS, CRH is responsible for stimulating adrenocorticotropin hormone (ACTH) release from the anterior pituitary, which allows the production of corticosteroids by the adrenal glands [3]. Therefore, when secreted properly, hypothalamic CRH acts in an anti-inflammatory fashion by producing cortisol, an anti-inflammatory steroid hormone. On the other hand, immune cells such as T lymphocytes and mast cells are responsible for the peripheral production of CRH in the adrenal medulla, testes, ovaries, cardiovascular system, gastrointestinal tract, pancreas, lung, and endometrium. It has been demonstrated that peripheral CRH is involved in inflammation by inducing histamine release via mast cell degranulation [4].

CRH receptors (CRH-R) belong to the G-protein coupled receptor superfamily. CRH acts by binding to CRH receptor type 1 (CRH-R1) and type 2 (CRH-R2), with a 10-fold higher affinity for the CRH-R1 versus CRH-R2 [5]. In addition, CRH-R1 binds urocortin (Ucn) with approximately equal affinity, whereas CRH-R2 binds Ucn, Ucn-2, and Ucn-3 with significantly higher binding affinity than CRH [1]. CRH-R1 activation by CRH or Ucn can stimulate macrophages and mast cells to release vascular endothelial growth factor (VEGF), tumor necrosis factor alpha (TNF-α), interleukin 1 beta (IL-1β), and interleukin 6 (IL-6), which are involved in the pro-inflammatory process. Conversely, experiments have revealed that CRH-R2 may have beneficial roles in inflammation, but these are dose- and time-dependent [6].

Early animal studies confirmed that stress increases hypothalamic CRH secretion [7–9]. The elevation of CRH in response to stress increases cortisol to maintain adequate blood glucose, blood pressure, renal function, and electrolyte distribution [10]. However, in stress-related diseases such as depression and anxiety, CRH is secreted excessively, hyper-activating the HPA axis and causing a detrimental physiologic state [11]. In this context, early studies started using CRH antagonists to investigate the physiologic and pathophysiologic roles of endogenous CRH in animals [12,13]. To our knowledge, in 1984, Rivier et al. performed the first preclinical study using a CRH antagonist -α-helical CRH 9-41- as a treatment for CRH-induced hypersecretion of ACTH in a rat model. They found that pretreatment with α-helical CRH 9–41 (a competitive CRH-R2 antagonist) significantly decreased plasma ACTH levels and inhibited the CRH-induced secretion of ACTH in a dose-dependent manner. In 1996, Baram et al. performed the first clinical trial administering α-helical CRH 9–41 to healthy subjects to assess the safety and hormonal efficacy of the antagonist [14]. The results revealed that the CRH antagonist reduced ACTH and cortisol levels without generating blood pressure, glucose, or electrolyte imbalances [14]. These findings suggested that CRH antagonists can be used to effectively treat stress-related disorders that disrupt homeostasis of the HPA axis. As a result, most pre-clinical and clinical trials to date concentrated on disorders of the central nervous system such as anxiety, depression, post-traumatic stress disorder and addictions, but this has been met with little success [15]. Importantly, deregulation of the HPA axis also strongly influences the gastrointestinal, reproductive, and immune systems, yet less attention has been placed on the possible therapeutic effects of CRH antagonists in these systems. *The overall goal of this review was to systematically synthesize the available evidence for the use of CRH antagonists (regardless of receptor specificity) for disorders associated with abdominal and pelvic organs*. As such inflammatory bowel disease (IBD) and irritable bowel syndrome (IBS) were expected to be some of the disease models used in animal studies to examine the effectiveness of CRH antagonists. IBD is a chronic gastrointestinal (GI) tract disease characterized by abdominal pain, weight loss, bloody stools, and diarrhea. Clinically, IBD is subdivided into Crohn’s disease, which affects any part of the GI tract, and ulcerative colitis, which affects only the colon. IBD pathogenesis is related to genetic, lifestyle, and environmental factors [16]. Worldwide, an increasing incidence of IBD has been observed over the past few decades; therefore, numerous therapeutic advances have emerged (e.g., [17,18]). However, current treatments generate many side effects and do not effectively cure this refractory disease [19].

IBS is a chronic GI disorder affecting the small and large intestines and is characterized by diarrhea, abdominal pain, bloating, flatulence, and constipation [20]. IBS pathogenesis includes GI motility dysfunctions, malabsorption, and gut microbial and enteric nervous system alterations. Even though its etiology remains unclear, evidence suggests that economic, sociologic, and psychological factors are involved [21]. Studies have shown that IBS affects the gut-brain axis, linking conditions like depression and anxiety to the disease, making its treatment more complex [22]. IBS therapy is currently focused on controlling the symptoms but is often ineffective or not tolerated by patients [23].

Preclinical studies have reported that peripheral CRH is implicated in the pathophysiology of abdominal and pelvic diseases such as IBD, IBS, endometriosis, and bladder disorders [24–27]. Therefore, this literature review summarizes the current pre-clinical evidence for CRH antagonists as a treatment in abdominal and pelvic diseases and discloses some of the barriers preventing these drugs from reaching the commercial arena.

## Methods

This is a Systematic review following the Preferred Reporting Items for Systematic Reviews and Meta-Analyses (PRISMA) statement. Articles were extracted from PubMed and Cochrane Library electronic databases. JEPB and JLC extracted the articles during the month of June 2021. Articles included in this study must have been indexed in the afore-mentioned databases from June 1, 2006—June 1, 2021 (15 years).

### Search strategy

The key words and Boolean searches used to identify articles were (1) Corticotropin releasing factor antagonist AND treatment, (2) Corticotropin releasing factor antagonist AND inflammation, (3) Corticotropin releasing hormone antagonist AND treatment, (4) Antalarmin AND treatment, (5) pexacerfont AND treatment, (6) Astressin AND treatment, (7) corticotropin releasing hormone antagonist AND inflammation, (8) verucerfont AND treatment, (9) CP-316,311 AND treatment, (10) NBI-30775 AND treatment, (11) NBI-34041 AND treatment, (12) ONO-2333Ms AND treatment, (13) emicerfont AND treatment, (14) crinecerfont AND treatment, (15) GSK-586529 AND treatment, (16) tildacerfont AND treatment, (17) SSR-125543 AND treatment, (18) NBI-74788 AND treatment, (19) GW-876008 AND treatment and (20) SPR001 AND treatment. To eliminate repeated articles between searches, conditional formatting for duplicate values using the PubMed ID was applied using Microsoft Excel.

### Research question

The following research question was formulated as the framework for the study: “What are the effects of corticotropin-releasing hormone antagonists when used in chronic disease models of the abdominal and pelvic organs?”

### Inclusion and exclusion criteria

Articles were included for this review if they were:

1. Studies in animals (pre-clinical) or any type of *in vivo* testing done in any animal model was included in the manuscript.
2. CRH-R1 and/or CRH-R2 antagonists used as a treatment or experimental tool in abdominal and pelvic organs.
3. Written in the English language.
4. Published between June 1, 2006, and June 1, 2021.

Articles were excluded from this review if they:

1. Focused exclusively on CRH antagonist synthesis and structure.
2. Assessed depression and anxiety without any observation of abdominal or pelvic organs.
3. Studied drug addiction and food craving exclusively, without reported effects on abdominal or pelvic organs.
4. Comprised *in vitro* studies exclusively.
5. Were qualitative research studies.
6. Were case reports, review articles or commentaries.
7. Assessed CRH agonist treatment.

For informative purposes, clinical studies were summarized in a table format and discussed in relevance to pre-clinical findings.

### Quality assessment

The Animal Research: Reporting of In Vivo Experiments guidelines 2.0 (ARRIVE) was used [28,29]. The ARRIVE Essential 10 is a checklist that uses ten items to evaluate study design, sample size, inclusion and exclusion criteria, randomization, blinding, outcomes measures, statistical methods, experimental animals, experimental procedures, and results. A modified methodology for quality assessment by Garcia-Gonzalez et al. [30]. was used to evaluate the articles. A scoring of 1 was given if the article met the required detail or recommendation, and 0 if not. The total number obtained by each article is reported in the corresponding table. The third reviewer (ATR) confirmed the first two reviewers (JEPB and JLC) scoring. The maximum score a study could receive was 21 points since some items in the ‘Essential 10’ checklist had more than one parameter that needed to be quantified. Since the review search dates pre-date the original publication of the ARRIVE guidelines, we carried out a sub-analysis of studies published on or before 2010 compared to those published from 2011–2021. Quality of the clinical studies was not assessed as these were only included for informative purposes.

### Parameters of interest

Elements collected from articles were first author, year of publication, animal model used (mice or rat), sex, testing compound, country where the study was done, and the main findings of the testing compound used in the study. The first two authors of this study carried out the data extraction. The mean difference between outcomes was not the focus of this review but rather the qualitative effect of the drug in the outcomes of interest. Therefore, the data extractors looked for specific terms in the results indicating: increase, decrease or no change; significant or not significant. In addition, the data extractors also noted when mechanisms of action related to the drugs were reported. These elements are presented in table format.

### Data synthesis

Articles were randomly divided into two sets evaluated by JEPB and JLC and systematically screened for inclusion criteria followed by exclusion using a table format in Microsoft Excel. Articles meeting all criteria for screening were then tabulated in a separate spreadsheet and the full extraction process began. The process was verified by the corresponding author to account for missing information. Since the ARRIVE guidelines were used for assessing the articles quality, the percent agreement between the scorers was calculated as well as the Spearman-Brown coefficient for the effectiveness of the “Essential 10” list for ARRIVE as applied to the articles included herein.

### Risk of bias

Three elements of the ARRIVE guidelines concern study biases: recommendation number three is related to the adequate reporting of inclusion and exclusion of experimental subjects; recommendation number four requires the reporting of randomization and strategies to minimize confounders; and recommendation number five requires the reporting of the tools used for blinding across the experiments. Failure of reporting across these three recommendations was considered a biased study.

### Statistical analyses

Descriptive analyses for the parameters of interest included mean, standard deviations, and percentages, as appropriate. Microsoft Excel was used to organize and summarize data elements. Statistical analyses and graphs were done using GraphPad Prism (v. 8, San Diego, California).

## Results

### Search results

**Fig 1:**
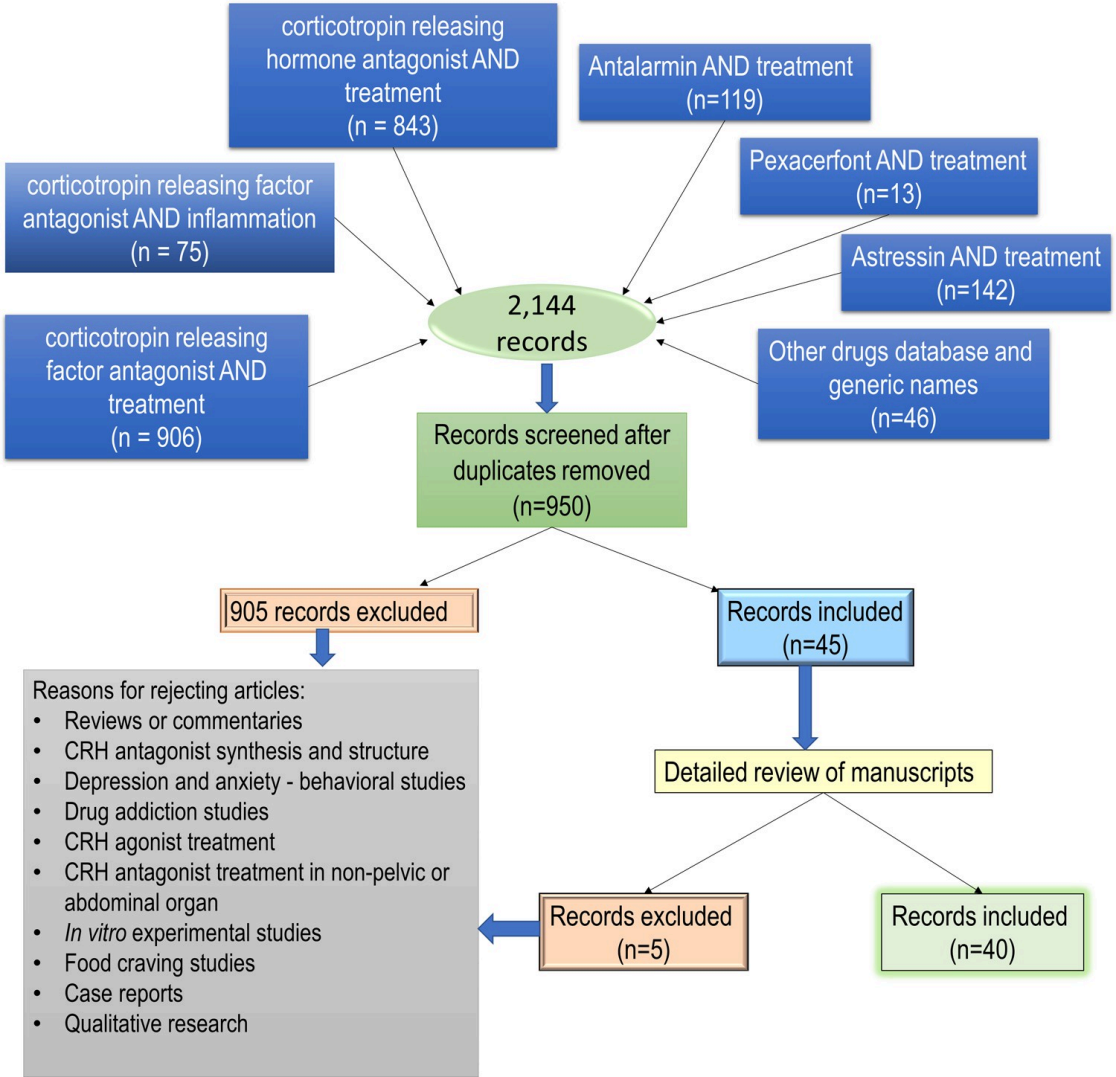
Article search results. Diagram depicting the number of articles found for the search strategies as established by the PRISMA guidelines.

**Fig 2:**
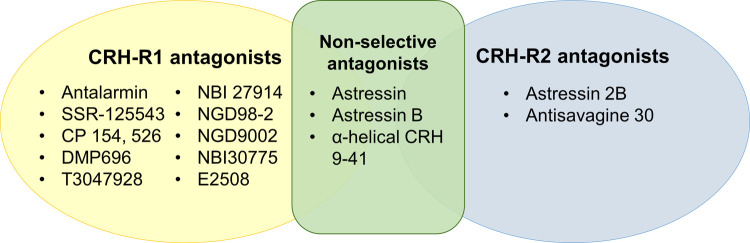
Compounds reported in the manuscripts reviewed. Ten compounds were used as CRH-R1 selective antagonists, while only two compounds were used as CRH-R2. Three non-selective CRH-R antagonists were reported in the studies.

Using the key word search strategy in PubMed, an initial 2,144 articles were generated from June 1, 2006, to June 1, 2021. Duplicates were eliminated resulting in 950 unique articles. Because of this, the following key words searches became redundant: corticotropin releasing hormone antagonist AND inflammation, verucerfont AND treatment, CP-316,311 AND treatment, NBI-30775 AND treatment, NBI-34041 AND treatment, ONO-2333Ms AND treatment, emicerfont AND treatment, crinecerfont AND treatment, GSK-586529 AND treatment, tildacerfont AND treatment, SSR-125543 AND treatment, NBI-74788 AND treatment, GW-876008 AND treatment, and SPR001 AND treatment. The resulting 950 article abstracts were screened, and 905 did not meet the inclusion criteria established. The 45 articles initially included went through a second screening round and only 40 met the inclusion criteria (**Fig 1**. Flowchart of search strategy). To study the beneficial effects of CRH-R antagonists different animal models were used. For this review, results will be presented by the type of disease model. This summary strategy was decided *a posteriori*, based on the obtained results. A total of six articles used CRH administration, 17 used stress models to observe gastrointestinal effects, 13 used administration of chemicals to induce inflammation, and four used surgical procedures to model the disease under study. **Tables 1–4** provide details and specific descriptions of the manuscripts included. Compounds used as antagonist were classified by their receptor selectivity (**Fig 2.** Diagram of compounds by receptor selectivity). As supplementary information, S1–S4 Tables include additional details of each study such as animal body weight (when reported), specific drug concentrations, and the type and duration of stressors in S2 Table.

**Table 1:** CRF administration models.

Author	Year	Animal model	Tested compounds	Country	Main findings	Quality Score
Ataka, K. et al.	2007	Rat model (Males)	Astressin, Antisavagine-30, NBI-27914	USA	Increased frequency of phase III-like contractions in colon, fecal pellet output and motor index % (MI%) induced by ICV injection of CRF was blocked by ICV injection of Astressin as well as by NBI-27914. However, it was not altered by Antisauvagine-30 (a selective CRF type 2 receptor antagonist).	12
Gourcerol, G. et al	2011	Rat and mouse model (Males and Females)	Astressin 2B, Urocortin 2	USA	Astressin-2B exacerbated CRF-induced fecal pellet output and diarrhea. Urocortin 2 decreased fecal pellet output induced by exogenous CRF administration	12
Million, M.** **et al.	2013	Rat model (Males)	NGD 98–2, NGD 9002	USA	NGD 98–2 or NGD 9002 SC pretreatment reduced the ICV and IP CRF-induced increase in fecal pellet output by 71% for each compound. However, when CRF was administered IP, only NGD 9002 significantly reduced FPO. NGD 98–2 and NGD 9002 oral pretreatment prevented the incidence of diarrhea induced by CRF. PO pretreatment of NGD 98–2 effectively reduced centrally or peripherally injected CRF- or acute water avoidance stress-induced colonic motor stimulation and repeated tonic colorectal distension-induced visceral hypersensitivity, while having no effect on defecation in a non-stressed rat.	13
Teitelbaum A et al.	2008	Rat model (Males)	Stressin I, Urocortin III and Antisauvagine	Canada	Antisauvagine inhibited chronic CRF administration induced colon permeability. CRF acted via mast cells to promote gut barrier dysfunction. Stressin1 increased secretory state mediated by mast cells, while urocortin III increased secretion and permeability in colon.	14
Tsukamoto, K.** **et al	2006	Rat model (Males)	Astressin	USA	Following IC pretreatment of Astressin (10 μg), the stimulatory effect on colonic motility of CRF was significantly attenuated to 29% of motor index change, compared with that of saline-injected rats of 35% (not significant) MI change.	9
Zhao, YX et al.	2021	Rat model (Males)	CP-154526 and Astressin 2B	China	Neither CP-154526 nor Astressin-2B alone exhibited any obvious effect on jejunal motility. Pretreatment with Astressin-2B significantly reversed the inhibitory action of CRF-induced jejunal motor index, but CP-154526 did not show any alleviating effect.	14

Abbreviations: Motor index % (MI%), intracerebroventricular (ICV), Corticotropin releasing factor (CRF), intraperitoneal (IP), Fecal pellet output (FPO), intracisternal (IC).

**Table 2:** Stress models.

Author	Year	Animal model	Testing compounds	Country	Main findings	Quality Score
Yoshimoto S. et al.	2011	Rat model (Males) Heterotypic stress	Astressin 2B, NBI-27914 and [d(CH2)1/5,Try(Me)2,Orn8]-Oxt	USA	NBI-27914 but not astressin2B inhibited the accelerated colonic transit by chronic heterotypic stress. Oxt antagonist reversed the restored colonic transit after chronic homotypic stress.	13
Barreau F. et al.	2007	Rat model (Males) Maternal deprivation	α-helical CRF 9–41, SSR-125543 and Doxantrazole	France	α-helical CRF 9–41 and SSR-125543 treatment decreased gut paracellular permeability induced by neonatal maternal deprivation. Doxantrazole decreased exogenous CRF increase in gut paracellular permeability.	13
Li B et al.	2017	Mouse model Maternal separation	Astressin, Astressin 2B and Antalarmin	Canada	Increases in IL6, TNF-α and iNOS were inhibited by pretreatment with Astressin and Antalarmin. However, Astressin-2β did not prevent the maternal separation-induced elevation in pro-inflammatory cytokines. Pretreatment of Antalarmin and Astressin during maternal separation improved colonic morphology, increased crypt length and the number of goblet cells per crypt, and decreased MPO (pro-inflammatory enzyme) levels. However, pretreatment with Astressin-2β did not rescue the MS-induced colonic injury and demonstrated no effect on immune activation, colonic morphology, and trans-cellular permeability. NF-κB (important protein complex in initiating and regulating inflammation in IBD) ratio of phosphorylated (active) to total NF-κB was higher in the MS group compared to controls, and was rescued by Antalarmin, but not Astressin or Astressin-2β.	13
Van den Wijnguard *et al*.	2012	Rat model (Males) Maternal separation	α- helical CRF 9–41 and Doxantrazole	Belgium	CRF antagonist as a pretreatment but not as a treatment prevented the visceral hypersensitivity induced by maternal separation and water avoidance in rats. CRF antagonist as a pretreatment had higher occludin levels.	14
Boucher W. et al.	2009	Mice model (Females) Restraint stress	Antalarmin and Astressin 2B	USA	Astressin 2B but not antalarmin decreased VEGF release from bladder after acute restrain stress. CRHR2 -/- and mice with double knock out for CRHR1-/- and CRHR2-/- had similar bladder vascular permeability after acute restrain stress compared to control.	11
Bülbül, M. et al.	2019	Rat model (Males) Restraint stress	Astressin	Turkey	Astressin pretreatment did not have any effect on acute restraint stress-induced changes in gastric emptying and intestinal transit. Astressin pretreatment recovered the CRF-induced deceleration of intestinal transit and gastric emptying completely. Astressin pretreatment prevented the inhibitory action of ARS on antral phase III-like contractions.	16
Gourcerol, G.** **et al.	2009	Mouse model (Males) Restraint stress	Astressin B, L-NAME and Atropine	USA	Acute restraint stress-induced increase in distal colonic contractions was blocked by the peripheral administration of Astressin-B. Astressin-B diminished the increase in fecal pellet output induced by restraint stress.	13
Kim D et al.	2010	Rat model (Males) Restraint stress	Astressin	Korea	Astressin decreased mast cells in proximal and distal colon of rat with total restraint stress. Astressin decreased proximal and distal protease activated receptors.	14
Nakade, Y. et al.	2007	Rat model (Males) Restraint stress	Astressin	Japan	IC Astressin pretreatment significantly abolished the restraint stress-induced acceleration of colonic transit. IP Astressin pretreatment did not affect restraint stress-induced acceleration of colonic transit.	12
Taguchi, R. et al.	2017	Rat model (Males) Restraint stress	E2508	Japan	Oral pretreatment of E2508 dose-dependently reduced wrap restraint stress-induced defecation. However, E2508 did not significantly inhibit basal defecation in non-restraint stress-induced rats. SC treatment of E2508 dose-dependently decreased the number of abdominal muscle contractions induced by colonic distention.	15
Bradesi, S. et al.	2008	Rat model (Males and Females) Water Avoidance	SSR149415 and DMP969	Sweden	DMP696 abolished visceral hyperalgesia induced by repeated water avoidance stress. DMP969 did not alter pain responses by colorectal distention. SSR149415 administration prevented VMR to colorectal distention after chronic water avoidance stress.	15
Buckley, M. et al	2014	Rat model Open field	Antalarmin and Monoclonal Anti IL-6 receptor	Ireland	Monoclonal anti IL-6 receptor co-administered with antalarmin synergistically decreased fecal pellet output. Anti IL-6 receptor and antalarmin increased pain threshold by colorectal distention.	16
Funatsu et al.	2007	Rat model (Males) Footshock	α- helical CRF 9–41, Loperamide, Ramosetron, Colansetron and Alosetron	Japan	α- helical CRF 9–41 reduced foot shock conditioned stress induced freezing behaviour and defecation while loperamide only decreased defecation. Ramosetron, cilansetron, and alosetron decreased defecation but not freezing behavior.	13
Robbins, M. et al.	2008	Rat model (Females) Footshock	aSVG30, Antalarmin and Urocortin 2	USA	Footshock treatment increased bladder hypersensitivity. aSVG30 but not antalarmin decreased footshock induced bladder hypersensitivity. Urocortin 2 provoked greater bladder hypersensitivity in naïve rats.	12
Seki, M. et al.	2019	Rat model (Males) Footshock	Antalarmin, Carbachol and CRF	Japan	Psychological stress increased muscarinic contractions in the bladder, which were partially prevented by Antalarmin daily treatment. Voided volume and micturition frequency per day were significantly lower in the psychological stress rats treated daily with Antalarmin than in rats psychologically stressed without any treatment. Mean voided volume per micturition was significantly higher in psychological stress rats treated daily with Antalarmin than in rats psychologically stressed without any treatment.	13
Itomi, Y. et al.	2020	Rat model (Males) Conditioning Fear stress	T-3047928, oCRH and Alosetron	Japan	T-3047928 pretreatment via oral route suppressed increased fecal pellet output induced by conditioning fear stress. However, 1mg of T-3047928 had no effect. Orally administered alosetron significantly reduced fecal pellet number in non-stressed rats. However, there was no impact on normal defecation when T-3047928 was orally given. T-3047928 pretreatment (3 and 10mg) improved both defecation and visceral pain in acute and chronic stress-induced disease models with similar dose range without causing constipation phenotype.	12
Roemer, E. J. et al.	2016	Mouse model (Male and Female) CRF Overexpression and acute stressors	Astressin B and Urocortin 2	USA	Astressin B pretreatment prevented the significant increase in fecal pellet output of female CRF-overexpressing mouse stress model compared with their wild type when exposed to a novel environment stressor. Astressin B pretreatment SC prevented the increase in fecal pellet output induced by partial restraint stress and exposure to a novel stressor, compared with control. Astressin B pretreatment had no effect on the number of urine spots (voiding response) induced by a novel environment stressor in female CRF-overexpressing mice.	12

Abbreviations: Oxytocin (OXT), Corticotropin releasing factor (CRF), myeloperoxidase (MPO), Maternal separation (MS), Vascular endothelial growth factor (VEGF), Acute restraint stress (ARS), Intra-cisternal (IC), intraperitoneal (IP), subcutaneous (SC), visceromotor responses (VMR).

**Table 3:** Chemical models.

Author	Year	Animal model	Testing compounds	Country	Main findings	Quality Score
Im, E. et al.	2011	Mouse model (Male and Female) DSS	Astressin 2B and Antalarmin	Korea	DSS-induced colitis mortality was decreased in mice injected IP daily with Antalarmin but increased in mice treated with Astressin-2B, compared with the vehicle-treated group. Antalarmin treatment flattened DSS-induced colitis weight loss, whereas Astressin-2B treatment accelerated weight loss. Histological damage analysis of the colon showed that the Antalarmin group had lower histological scores and the Astressin-2B group higher histological scores compared with the vehicle group.	12
Gong S. S. et al.	2018	Mouse model (Males) DSS	Astressin 2B and Urocortin 2	China	Compared with the DSS induced-colitis vehicle treated-group, mice treated daily with Ast2B showed more body weight loss, shorter colon lengths, significantly higher disease activity index (DAI) scores and histological scores. However, Urocortin 2 treatment improved these variables. Daily Astressin2B treatment increased protein expression levels of pro-inflammatory factors (TNF-α, CXCL-1 and IL-6) and TUNEL positive cells (increased apoptosis), and decreased cell proliferation. However, Urocortin 2 treatment showed the opposite effects. Intestinal permeability was higher in Astressin2B treatment compared with Urocortin 2 treatment in DSS-induced colitis rats.	16
Hoffman, J.M. et al.	2016	Mouse model (Males) DSS	Astressin 2B	USA	Histological analysis revealed that DSS-induced colitis mice treated daily with Astressin2B had more severe colitis, a decrease in body weight %, and a higher overall damage score (crypt damage, leukocyte infiltration and epithelial regeneration) than vehicle-treated controls. Colonic TNF-α, CXCL-1, and IL-6 mRNA levels, and TNF-α and IL-6 protein expression levels were elevated in mice treated with intracolonic Ast2B, whereas the anti- inflammatory cytokines IL-4, IL-5, and IL-12 remained unchanged. TUNEL positive cells (estimates the number of cells with DNA damage) increased by 55% in Asressint2B-treated mice compared with vehicle controls, indicating increased levels of apoptosis.	13
Jia, F. et al.	2013	Rat model (Males) Acetic Acid	α- helical CRF 9–41, NBI-27914 and Anti nsfatin 1/NUCB2	China	α- helical CRF 9–41, NBI-27914 and anti-nesfatin1/NUCB2 decreased visceral sensitivity in neonatal acetic acid rat model.	15
La, JH. et al.	2008	Rat model (Males) Acetic Acid	Astressin	Republic of Korea	Increased peripheral CRF promoted post inflammatory visceral hypersensitivity. Astressin reduced post inflammatory visceral hypersensitivity	12
Kokkotou, E. et al.	2006	Mice model (Males) Toxin A	Astressin 2B	USA	Astressin-2B prevented *C*. *difficile* toxin A induced enteritis by decreasing epithelial damage, immune cell infiltration and chemokines.	13
Kubo, Y. et al.	2010	Rat model (Males) Indomethacin	NBI-27914, Astressin2B, Astressin and Urocortin I	Japan	Subcutaneously administered indomethacin produced multiple hemorrhagic lesions in the small intestine which were aggravated by Astressin-2B and Astressin pretreatment. However, the selective CRH-R1 antagonist NBI-27914 had no effect on the severity of the lesions. Neither Astressin-2B nor NBI-27914 alone had any effect on the intestinal hypermotility response to indomethacin.	10
Larauche, M. et al.	2009	Mouse and rat models (Males) Cortagine	Astressin B and CP154,526	USA	CP-154,526 pretreatment prevented cortagine-induced defecation by 85% and abolished the diarrhea in rats. Cortagine-induced increase in colonic permeability was blocked by IV Astressin-B pretreatment. Cortagine injected IP increased the fecal pellet output, colonic permeability and motility, visceral hyperalgesia to phasic colorectal distension and induced diarrhea in 60% of mice (IBS model).	15
Liu, L. et al.	2011	Rat model (Males) Iodoaceta-mide	Antalarmin	USA	Neonatal gastric irritation induced depression and anxiety behaviour which was reversed by antalarmin administration.	14
Nakade, Y. et al.	2007	Rat model (Males) GLP-1	Astressin	USA	ICV injection of Astressin itself did not modify colonic transit. ICV Astressin pretreatment abolished ICV GLP-1-induced acceleration of colonic transit.	13
Nozu T. et al.	2014	Rat model (Males) LPS	Astressin 2B and Urocortin 2	Japan	Astressin-2B pretreatment did not modify the reduced motor index change or the suppressed antral contractions induced by LPS. Urocortin 2 completely disinhibited LPS-induced suppression of antral contractions. Administration of Astressin-2B, immediately before intraperitoneal Urocortin 2 administration, completely blocked Urocortin 2 disinhibitory effect on LPS-induced suppression of antral contractions.	13
Nozu, T. et al.	2019	Rat model (Males) Water Avoidance	Astressin 2B	Japan	Astressin-2B pretreatment did not modify the increased colonic permeability induced by LPS. Astressin-2B reversed DHEA-S effects on visceral allodynia and colonic permeability.	13
Saito-Nakaya, K. et al.	2008	Rat model (Males) TNBS	CP154,526	Japan	CP-154,526 attenuates visceral hypersensitivity induced by TNBS previous inflammation.	12

Abbreviations: Dextran sulfate sodium (DSS), intraperitoneal (IP), Disease activity index (DAI), intravenously (IV), Glucagon like peptide 1 (GLP-1), Lipopolysaccharide (LPS) and 2,4,6-trinitrobenzene sulfonic acid (TNBS).

**Table 4:** Surgical models.

Author	Year	Animal model	Testing compounds	Country	Main findings	Quality Score
Grandi. D. et al.	2008	Rat model (Males) Adrenalectomy	α- helical CRF 9–41, Mifepristone and Neuropeptide nociception/orphanin FQ (N/OFQ)	Italy	Healing effect of N/FOQ on gastric mucosa damaged by adrenalectomy or ethanol was not affected by α- helical CRF 9–41 or mifepristone.	10
Torres Reveron, A. et al.	2018	Rat model (Females) Endometriosis	Antalarmin	PR and USA	Antalarmin administration during the 7 days after endometriosis induction resulted in a 30% significant decrease in the number of developed endometriosis vesicles at 60 days. The total weight of endometriosis vesicles in the Antalarmin treated group was 67% less than the vehicle control group. (The reduced weight was a direct result of the smaller size of the vesicles in average volume and area per rat)	16
Takeuchi, K. et al.	2016	Rat model (Males) Enteritis	NBI27914, Astressin, Astressin-2B and Urocortin I	Japan	Ischemia induced by clamping the superior mesenteric artery followed by reperfusion caused hemorrhagic lesions in the small intestine, which were reduced dose-dependently by pretreatment with Astressin (3–30 μg/kg, IV) and Astressin-2B (60 μg/kg, IV), with a significant inhibition of lesions of 71.1% and 72.4% respectively. However, NBI-27914 had no effect. The aggravating effects of Urocortin I in I/R-induced intestinal lesions and in the increase of myeloperoxidase activity (> inflammation) in the small intestinal mucosa were significantly abrogated by the co-treatment with Astressin-2B, but not by NBI-27914. Pretreatment (before ischemia) and posttreatment (after reperfusion) with Astressin-2B significantly suppressed enhancements in bacterial invasion in the intestinal mucosa and the up-regulated expression of iNOS on the small intestine following I/R, while that of NBI27-194 did not.	12
Wood, S. K. et al.	2013	Rat model (Males) Partial bladder outlet obstruction	NBI-30775 and Tartaric acid (vehicle)	USA	NBI-30775 SC pretreatment significantly prevented the increase of all urodynamic measurements (intermicturition interval, micturition volume and bladder capacity) stress-induced urodynamic dysfunction but had no effect in control rats. NBI-30775 SC pretreatment significantly prevented the increase of all urodynamic measures in partial bladder outlet obstruction-induced urodynamic dysfunction. However, NBI-30775 did not reduce the increased micturition pressure	13

Abbreviations: Ischemia reperfusion (I/R) and subcutaneous (SC).

### Models

#### CRH administration model

To increase colonic motility intracerebroventricular (ICV) administration of CRH in male rats was done by Ataka et al. which resulted in increased fecal pellet output (FPO) and motor index. In their study, administration of astressin (non-selective CRH antagonist) and NBI-27914 (CRH-R1 selective antagonist) inhibited the afore mentioned effects of CRH but antisauvagine-30 (selective CHR-R2 antagonist) did not [31]. Gourcerol et al. observed that administration of astressin 2B (CRH-R2 antagonist) exacerbated CRH induced FPO and diarrhea while urocortin 2 decreased these effects [32]. In the study by Million et al. pretreatment with NGD 98–2 or NGD 9002 (both CRH-R1 antagonists) reduced the ICV CRH induced FPO, but when CRH was administered by IP injection, only NGD 9002 reduced FPO [33]. On the other hand, Teitelbaum et al. observed that administration of antisauvagine inhibited chronic CRH induced colon permeability [24]. In the study of Tsukamoto, et al. pretreatment of astressin attenuated CRH induced colonic motility in male rats [34]. Finally, Zhao Y. and colleagues using male rats observed that pretreatment with astressin 2B, but not CP-154526 (selective, non-peptide CRH-R1 antagonist) reversed the inhibitory action of CRH on jejunal motor index [35]. See Table 1 for additional details.

#### Stress models

In the study by Yoshimoto, S. male rats were exposed to chronic heterotypic stress (water avoidance, forced swim stress, cold restraint stress and restraint stress) and treated with NBI-27914 and astressin-2B, but only NBI-27914 inhibited the accelerated colonic transit [36]. On the other hand, Barreau et al., observed that male rats with induced stress by neonatal maternal deprivation exhibited increased gut paracellular permeability whereas treatment with α-helical CRH 9–41 and SSR-125543 (selective CRH-R1 antagonist) decreased this effect [37]. In the study of Li B., mice exposed to maternal separation were observed to have increased IL-6, TNF- α and inducible nitric oxide synthase (iNOS), which were inhibited by pretreatment with astressin and antalarmin (CRH-R1 antagonist) [26]. However, astressin 2B did not prevent the afore mentioned effects. In addition, antalarmin and astressin improved colonic morphology and decreased myeloperoxidase enzyme; however only antalarmin was able to decrease phosphorylated NF-kB. Van den Wijnguard observed that administration of α-helical CRH 9–41 as a pretreatment, but not as a treatment, prevented visceral hypersensitivity induced by maternal separation and water avoidance in male rats [38]. In addition, animals receiving the antagonist pretreatment had higher occludin levels. In another study, Boucher et al., demonstrated that astressin 2B but not antalarmin decreased VEGF release from bladder after acute restraint stress in female mice [39]. Bulbul et al. observed that although astressin pretreatment didn’t have any effect on acute restraint stress-induced changes in gastric emptying and intestinal transit, astressin pretreatment recovered the exogenous CRH induced deceleration of intestinal transit [40]. Other studies that used restraint stress observed that astressin-B decreased FPO and blocked the increase in distal colon contractions [41], and astressin decreased proximal and distal colonic mast cells [42]. However, intracisternal administration of astressin was observed to abolish restraint stress-induced acceleration of colonic transit while IP administration was not effective [43]. In the study by Tagushi et al., oral pretreatment of E2508 (CRH-R1 antagonist) reduced defecation induced by restraint stress while subcutaneous (SC) administration as a treatment decreased abdominal muscle contraction due to colonic distention [44]. Furthermore, SC pretreatment of astressin-B prevented the increase in FPO induced by partial restraint stress, and similar results were observed in female CRH overexpressing mice with astressin-B pretreatment [45].

The water avoidance model was used by Bradesi et al. to induce psychological stress. Although DMP696 (CRH-R1 antagonist) abolished visceral hyperalgesia it did not alter the pain response caused by colorectal distention [46]. Similarly, Buckely et al. used an open field to induce psychological stress where coadministration of the monoclonal IL-6 receptor with antalarmin synergistically decreased FPO and pain threshold to colorectal distention [47]. On the other hand, three studies used footshock as a model to induce stress where Funatsu et al. observed decreased defecation and freezing behavior by α-helical CRH 9–41, Robbins et al., observed that administration of antisauvagine 30 but not antalarmin decreased bladder hypersensitivity and Seki et al., observed that antalarmin partially prevented muscarinic contractions [48–50]. Finally, conditioning fear stress was used by Itomi et al. where oral pretreatment with T-3047928 (CRH-R1 antagonist) suppressed the increase in FPO [51]. See Table 2 for additional details.

#### Chemical models

In preclinical studies administration of chemical compounds was mainly used to induce IBD or IBS-like symptoms in rodent models. Beginning with IBD models, three studies used dextran sulfate sodium (DSS) to induce colitis in male mice to investigate the effects of CRH antagonists. In the study by Im et al., daily IP injection with antalarmin lowered the histological damage found in the colon, decreased the mortality rate, and flattened the loss of weight caused by DSS-induced colitis [52]. Conversely, daily IP astressin-2B treatment resulted in higher histological damage scores of the colon, a raised mortality rate, and increased weight loss in DSS-induced colitis. Gong et al. demonstrated that daily IP astressin-2B treatment for DSS-induced colitis resulted in increased weight loss, higher disease activity index (DAI) scores, and histological damage scores [53]. In addition, astressin-2B treatment increased expression levels of pro-inflammatory cytokines (TNF-α, CXCL-1, and IL-6), increased cells with DNA damage, and increased intestinal permeability. Lastly, Hoffman et al. showed that daily IC treatment with astressin-2B for DSS-induced colitis resulted in increased weight loss, higher histological damage scores, and increased expression of pro-inflammatory cytokines [54]. Results also demonstrated that anti-inflammatory cytokine levels (IL-4, IL-5, and IL-12) remained unchanged, and the number of cells with DNA damage was increased.

Kokkotou, et al. injected *Clostridium difficile* toxin A in the gut of male mice to induce inflammation. Pretreatment with astressin-2B injected IP prevented *C*. *difficile* toxin A-induced enteritis by decreasing epithelial cell damage, immune cell infiltration, and inflammatory chemokines [55]. Similar results were obtained in CRH-R2 knockout animals. La, et al. used IC administration of acetic acid to induce colitis in male rats [56]. Results demonstrated that IP treatment with astressin reduced post-inflammatory visceral hypersensitivity reducing the abdominal withdrawal reflex (AWR) scores. Saito-Nakaya, et al. worked with an IBD model of colitis by injecting trinitrobenzene sulfonic acid (TNBS) into male rats [57]. SC treatment with CP-154,526 attenuated the increased visceral hypersensitivity caused by TNBS inflammation. In the study by Kubo, et al., male rats were injected with indomethacin to induce small intestinal lesions and bowel hypermotility [58]. Indomethacin-induced hemorrhagic lesions were aggravated by intravenous (IV) pretreatment of astressin-2B and astressin, but no effects were shown when using NBI-27914. Additionally, neither of the antagonists improved bowel hypermotility.

Referring to IBS models, Jia, et al. administered acetic acid to induce IBS-like visceral hypersensitivity in male rats [59]. ICV treatment with α-helical CRH 9–41 and NBI-27914 significantly decreased AWR scores and reduced the electromyographic activity in the external oblique muscle in response to colorectal distension. In an IBS model, CRH antagonists were used by Larauche, et al. to test the impacts of cortagine-induced stress-like alterations of colonic function and visceral hypersensitivity in male rats [60]. IV astressin-B pretreatment blocked the cortagine-induced increase in colonic permeability, and SC CP154,526 pretreatment abolished cortagine-induced diarrhea. Nakade, et al. used glucagon-like peptide (GLP) 1 to induce IBS-like acceleration of colonic transit in male rats [61]. Results demonstrated that ICV astressin pretreatment reduced GLP1-induced acceleration of colonic transit. Liu, et al. used iodoacetamide to induce gastric irritation and, consequently, depression in male rats [62]. Antalarmin administered orally reversed the depression-like behavior in these animals.

Nozu, et al. used lipopolysaccharide (LPS) administration in male rats to observe gut motility inhibitory effects [63]. Pretreatment with IP astressin-2B did not modify the reduced motor index change or the suppressed antral contractions induced by LPS. In 2019, Nozu et al. conducted another study using the same LPS rat model where they observed that astressin-2B also does not modify the increased colonic permeability induced by LPS [64]. Additional details for manuscripts in this section are presented in Table 3.

#### Surgical models

To understand the healing effect of nociceptin/orphanin FQ (N/FOQ), ethanol-induced gastric mucosa damage in adrenalectomized rats was induced by Grandi and colleagues. In their study, administration of α-helical CRH did not alter the N/FOQ healing effect on gastric lesions. In a study by our laboratory, we have shown that administration of antalarmin for 7 days in female rats with endometriosis surgery decreased the number and weight of vesicles developed [27]. In another study, Takeuchi, et al. induced enteritis in male rats by clamping the superior mesenteric artery which caused hemorrhagic lesions in the small intestine [65]. Here, pretreatment of astressin and astressin-B, but not NBI-27914 inhibited the lesions. Wood, et al. used social stress and partial bladder outlet obstruction to induce urodynamic dysfunction in male rats [25]. Pretreatment with NBI-30775 (CRH-R1 antagonist) administered SC prevented the increase of intermicturition interval, bladder capacity, and micturition volume induced by social stress. Similarly, daily treatment with NBI-30775 prevented the increase of all urodynamic measurements mentioned above caused by partial bladder outlet obstruction surgery. Table 4 provides additional details about the studies included in this section.

### Sex representation in the studies

**Fig 3:**
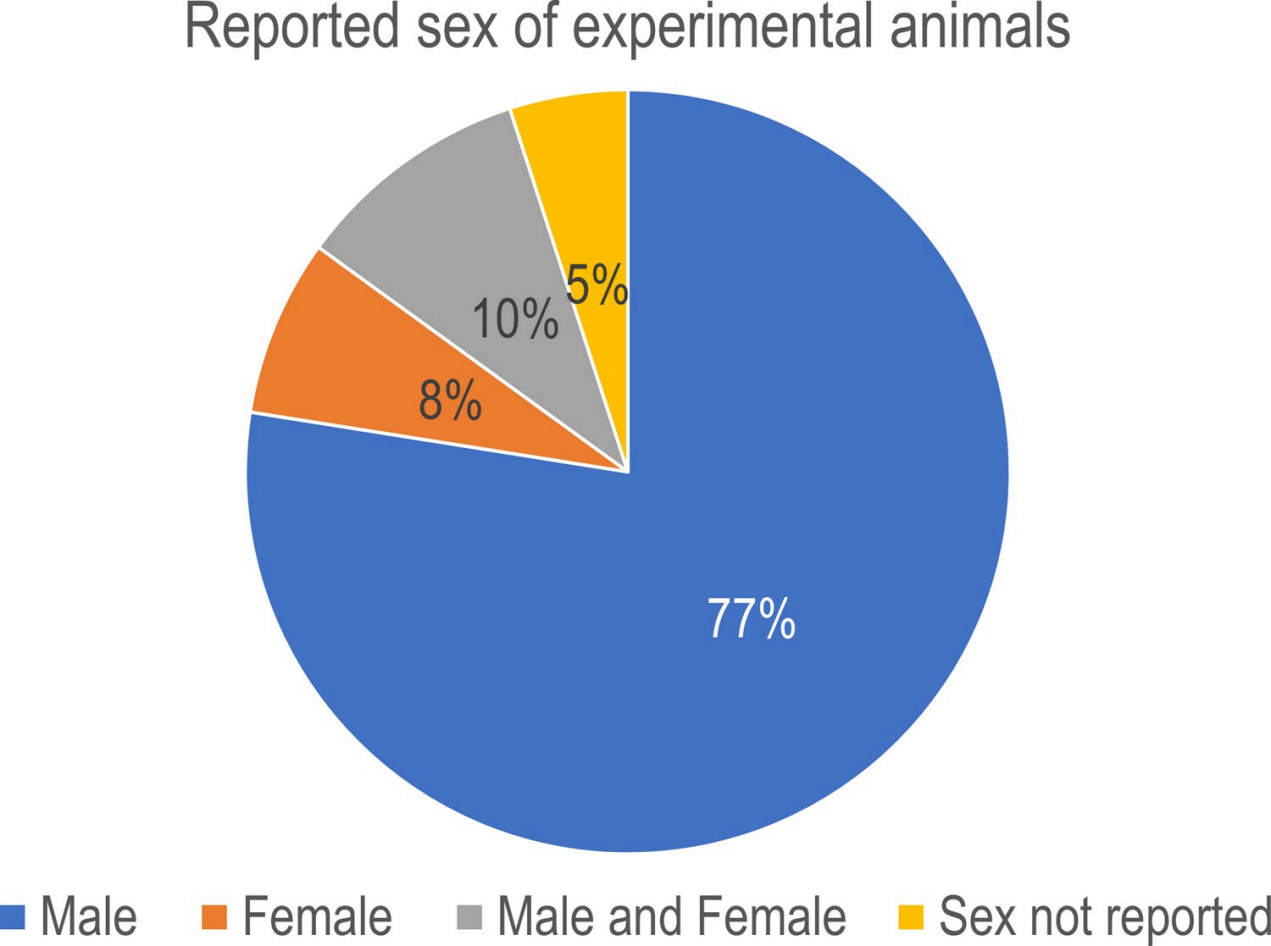
Sex distribution in the animal studies. Pie chart summarizing the percent of studies that included males, females or both sexes in their experiments. An over representation of males in the studies is evident.

From the six studies that used CRH administration as their experimental model, five used only males and one had both males and females. In the 17 studies that used stress as their experimental model, 11 were done in males, two in females, two had males and females and two did not specify sex. On the 13 studies using 10 different chemical models, only one study used male and female animals while the remaining 12 used males only. From the studies using surgical models, three used males and one study used females. Overall, 77% of studies included were done only in males, 8% in females, 10% in males and females, and 5% were unspecified (**Fig 3**).

### ARRIVE results

The average score for ARRIVE was 13.08 (SD 1.64) points. The group with the lowest score were the ones in the CRF administration group, presented in Table 1, with an average score of 12.3 points. The group of manuscripts with the highest average score were the ones using stress models, presented in Table 2, with an average score of 13.35. When the risk of bias was evaluated using the elements of inclusion and exclusion reporting, randomization, and blinding, 77% of all studies complied with at least one of the elements of inclusion and exclusion criteria reporting. Unfortunately, only 44% of the studies included some type of randomization of the animals and blinding was only reported in 41% of the studies. Of note, most studies blinded the investigators during scoring of tissues or analyses but did not mention any type of blinding related to animal testing.

Recognizing that some studies included in this review pre-date the original publication of the ARRIVE guidelines, a sub analysis of studies published on or before 2010 revealed an average of 10.93 (SD 1.98), while those studies published 2011 onward had a score of 13.26 (SD 0.47). This shows an average increase of only 2.35 points in the quality of the studies in a period of approximately 10 years (2011–2021). Finally, the percent of agreement within scorers was calculated at 82.1%. Since we used the Essential 10 recommendations for the ARRIVE guidelines, we also calculated the Spearman- Brown coefficient at 0.779, which is considered acceptable.

### Clinical studies

We found three clinical trials manuscripts that report data on CRH antagonists. All three manuscripts were Phase 2 studies (in the United States) and all three were done in women. Interestingly, while IBS was the target disease, two of them report the brain activity [66,67] while only one reports outcomes on gastrointestinal motility and abdominal pain [68]. **Table 5** summarizes the main findings of these studies for informative purposes only.

**Table 5:** Summary of clinical trials using CRH antagonists against disorders of the abdominal or pelvic area.

Author	Year	Clinical phase	Testing compound and dose	Main findings
Hubbard CS. et al.	2011	Phase II Women with an average age of 35 years	GW876008 20 or 200 mg	CRH-1 upregulation in the locus coeruleus complex attributable to chronic stress exposure in IBS patients was reduced by GW876008 administration, bringing levels of activation to that seen in control subjects. GW876008 administration did not affect plasma ACTH or cortisol levels in IBS patients or control subjects, but could attenuate stress-induced hypothalamic activation during expectation of abdominal pain. GW876008 resulted in significant blood-oxygen level-dependent activity reductions within regions of an emotional–arousal circuit during pain expectation in both patients and control subjects.
Labus J. et al.	2013	Phase II Women with an average age of 33 years	GW876008 20 or 200 mg	In IBS patients, the CRH-R1 antagonist produced greater suppression of blood-oxygen level-dependent activity in a wide range of brain regions during extinction of a conditioned fear response, and this inhibition was associated with a reduction of skin conductance response. During extinction, the effects of GW876008 on brain activity in response to a conditioned stimulus indicated greater suppression of pons and left midbrain in IBS compared to controls. During the acquisition phase of the conditioned fear response, GW876008 significantly suppressed lateralized clusters of activity in the thalamus in both patients and controls, and additionally suppressed midbrain activity in IBS patients.
Sweetser S. et al.	2009	Phase II Women 18–65 years-old	Pexacerfont 25 or 100 mg	Pexacerfont did not alter colonic transit in women with diarrhea predominant-IBS. No other meaningful effects were noted on gastric emptying, orocecal transit, and ascending colon emptying. There were no significant treatment effects detected by pexacerfont on the number of stools per day, ease of passage scores, stool consistency or subjective symptoms including bloating, gas, urgency, or abdominal pain.

## Discussion

In this review, the goal was to determine what effects corticotropin-releasing hormone antagonists have in chronic diseases of the abdominal and pelvic organs. Overall, the results demonstrated a beneficial response by blocking the CRH-R1, while blocking CRH-R2 was mainly associated with worsening the conditions under study. The timing of drug administration, route of administration, and the specific animal model used were seen to have an impact on the beneficial effects observed.

It was noted that the largest number of papers published for CRH receptor antagonists occurred in 2007 (150 papers in PubMed); hence the strategy of this review to cover a 15-year period. However, the rate of publication dropped more than 70% by 2020 indicating decreased research focusing on CRH receptor antagonists, most likely responding to the lack of positive results around mood disorders (as previously reviewed in [15]). Nevertheless, an assessment of CRH-R antagonists as therapeutic agents in peripheral disorders was absent and this review intended to fill that gap. Based on our findings, we propose that CRH-R antagonists still have a strong therapeutic potential in the context of HPA axis dysregulation for disorders outside the CNS.

### Interpretation of main findings

Rodents pretreated with CRH-R1 selective antagonists and consequently exposed to CRH administration, acute stress, or chemicals that cause GI irritation or dysfunction presented better disease outcomes than rodents pretreated with CRH-R2 selective antagonists. Among the CRH1 antagonists included in this review, antalarmin and NBI-27914 were the most frequently used, appearing in 15 and 6 of the preclinical studies, respectively. CRH1 antagonists demonstrated beneficial roles in treating stress symptoms, GI diseases, bladder disorders, and endometriosis. In stress models, CRH-R1 antagonists inhibited the increase of pro-inflammatory cytokines, myeloperoxidase levels, inflammatory transcription factors, colonic transit, FPO, and improved colonic morphology and urodynamic dysfunctions [25,26,36,47]. CRH-R1 antagonists decreased colonic lesion scores, mortality rate, weight loss, diarrhea, and depression-like behavior in GI disorders models [52,60,62]. In an endometriosis rat model, a CRH-R1 antagonist reduced the number and weight of endometriotic vesicles [27]. Therefore, blocking CRH-R1 confers a protective effect against inflammatory and stress-related diseases of the abdominal and pelvic organs.

CRH-R2 antagonist pretreatment commonly caused the opposite effects of CRH-R1 blockers, although there were a few exceptions. Referring to these studies, CRH-R2 antagonists reversed the CRH-induced increase in jejunal motility and colonic permeability and prevented *C*. *difficile* toxin A-induced enteritis as well as ischemia-induced hemorrhagic intestinal lesions, bacterial invasion, and macrophage inflammatory phenotype [24,35,55,65]. Otherwise, CRH-R2 receptor blockage was associated with increased pro-inflammatory cytokines, FPO, intestinal permeability, inflammatory transcription factors, weight loss, mortality rate, and colonic lesion scores [26,32,52,53].

Non-selective CRH antagonist use revealed a pivotal role between beneficial and detrimental effects, basically because they could block both CRH-R1 and CRH-R2 receptors. Most findings found more beneficial effects than harmful ones. However, in many studies, the non-selective CRH antagonists could not prevent stress-induced changes in gastric emptying and intestinal transit, reduced bowel hypermotility and inflammatory transcription factors, and improved GI hemorrhagic lesions induced by chemicals [26,40,58]. Since the primary goal for treating abdominal and pelvic diseases in rodents is to prevent the activation of CRH-R1 and stimulate CRH-R2 activation, CRH-R1 antagonists appear to be the drugs of choice.

### Quality of the studies

Outcomes from the quality assessment indicate a discrepancy between the clinical and preclinical scenario. IBS, IBD and gastrointestinal disorders are known to be twice as likely in females compared to males [69]. Yet the vast majority (77%) of the studies included herein were in male rodents only. Only 16% of these studies included female rodents to test the efficacy of CRH-R antagonists in chronic diseases. A lack of inclusion of both sexes in the pre-clinical studies represents a critical barrier in the current understanding of the beneficial effects of CRH-R antagonists for gastrointestinal disorders. Furthermore, a lack of pre-clinical data in females could pose a significant barrier as CRH receptors are present in reproductive tissues in abundance and are known to have a paracrine role in the uterus and ovaries [70]. Therefore, further studies should be inclusive with representation of both sexes, especially when the modeled disease is well known to be more frequent in females.

Close to 60% of the studies did not use any type of blinding or randomization which is worrisome. This could be explained by either a lack of compliance for these two essential elements during the experiments, or by a failure in reporting within the manuscripts. It is impossible to distinguish between these two elements, but they strikingly contrast with the ARRIVE guidelines, which were first introduced in 2010 and modified in 2019. While some of the studies included in this review pre-dated the publication of the guidelines used for evaluation, the field had a minimal increase in the quality of the studies reported over a decade. This suggests that either the investigators are ignoring essential elements for appropriately conducting animal studies, or that the ARRIVE guidelines have not had any significant impact on the field. Additional reviews will be needed to answer this important issue.

### Clinical relevance and recommendations

CRH-R1 antagonists have been extensively used in preclinical studies, demonstrating some positive effects for treating not only abdominal and pelvic diseases but also depression and anxiety. However, when studied in clinical trials, the results are not reproducible as seen in animals. Some of the limitations for clinical translation have been carefully reviewed by Spierling and Zorrilla [15] and include issues with pre-clinical screening, the dynamic nature of CRH receptor function not matching the tested patients, acute vs. late presentation of disease, dynamic brain plasticity of CRH receptor activity leading to decreased need for antagonism, among other factors. However, Spierling and Zorrilla emphasized that we still do not fully understand CRH role, especially related to brain disorders. This section explores some of the obstacles and flaws preventing CRH-R1 antagonists from reaching the commercial arena and their possible benefits over current treatments for chronic abdominal and pelvic diseases in humans.

IBS and IBD are chronic refractory diseases whose treatments are based on relieving symptoms to improve patient quality of life. The Mayo Clinic website and the Crohn’s and Colitis Foundation of America recommend treating these diseases with medication, a healthy diet, stress and pain management activities, and surgery for severe IBD cases [71]. Although current pharmacological treatments focus on relieving symptoms, they have many side effects and sometimes a limited impact on patient well-being [19,23]. For example, long-term use of corticosteroids for IBD can provoke osteoporosis, cataracts, and adrenal insufficiency [72], whereas antispasmodics for diarrhea-predominant IBS (D-IBS) can cause dizziness, blurred vision, xerostomia, and constipation [73].

Most preclinical studies in this review found that CRH-R1 antagonists can inhibit or diminish gastrointestinal symptoms and reduce stress-induced symptoms caused by CRH administration, perhaps by inhibiting CRH-R1 receptor-mediated inflammation. Further, testing in control rats indicates good safety profiles [37,43], and suggests that CRH-R1 antagonists may improve upon IBD and IBS current treatments by dealing with GI dysfunction and stress levels without the need for polypharmacy. Despite such findings, a path towards the commercial arena has not occurred. As previously pointed out, one of the limitations of the available preclinical studies is the predominant use of male animals when many of these diseases are far more frequent in women. This discordant design flaw might contribute to the lack of progress for these drugs in reaching the commercial arena for treating chronic abdominal and pelvic diseases.

Clinical trials to date include a Phase II trial to evaluate the pharmacodynamic effects of pexacerfont (BMS-562086), a CRH-R1 antagonist, in women with diarrhea predominant IBS [68]. Oral administration of pexacerfont had no significant impact on colonic transit changes, the number of stools per day, stool consistency, or subjective symptoms of bloating and urgency. The reasons for this apparent lack of efficacy on GI dysfunction are still unclear. Pharmacokinetic measurements demonstrated that the drug reached the desired plasma concentration, however there was no certainty that it was present in the GI tract where symptoms were exhibited. Although the oral route is more convenient for patients, it may delay drug effectiveness, reducing bioavailability [74]. Additionally, this trial was of short duration (14 days), perhaps insufficient to observe the effects of pexacerfont in this chronic condition. Encouragingly, 10% of study patients had minimal side effects that resolved without interventions. Pexacerfont also had no impact on subjective measures of emotion, a finding compatible with results from other clinical trials using the selective CRH-R1 antagonist, GW876008 [66,67]. These studies tested the efficacy of this drug in modulating the emotional-arousal circuit in women with IBS focusing on the CNS connections in the emotional-arousal circuit. The results showed that GW876008 could attenuate stress-induced hypothalamic activation during abdominal pain expectation and significantly reduced blood-oxygen-level-dependent activity within regions of an emotional–arousal circuit. Therefore, these drugs may be more effective in stress-sensitive patients showing disruptions in the HPA axis and suffering from comorbid mood disorders such as anxiety and depression.

Future clinical trials should focus on exploring the effectiveness of CRH1 antagonists, specifically antalarmin and NBI-27914, in reducing GI symptoms, improving long-term disease outcomes, and comparing these drugs’ advantages over current treatments for IBD and IBS. There are few preclinical studies of CRHR1 antagonists treating endometriosis and bladder disorders; therefore, additional studies must be done before these drugs reach clinical trials for these diseases such as the studies included in this review that demonstrated positive results [25,27,50]. Current evidence in this review supports that there is still hope for CRH-R1 antagonists to reveal their potential as emerging treatments for abdominal and pelvic chronic diseases.

### Conclusions

Current pharmacological treatments for IBS and IBD are based on improving disease symptoms but not actually curing these diseases, and unfortunately many treatments are known to cause adverse effects. Most of the preclinical studies identified during this review not only prove the effectiveness of CRH antagonists for the treatment of abdominal and pelvic diseases, but also demonstrate that these drugs have minimal side effects. CRH antagonists can potentially offer novel alternatives for patients with abdominal and pelvic pain. Still, more clinical trials should be undertaken focusing on the beneficial effects of these drugs in the peripheral organs given the abundance of CRH receptors in gastrointestinal and reproductive tissues. Although the failure of these drugs in past trials for mood disorders may have significantly decreased enthusiasm to continue pursuing a clinical application, re-focusing their expected outcomes on peripheral organs might offer an exciting avenue for future therapeutics.

## Registration and protocol

This review was not registered. The protocol used for this review is explicitly outlined in the methods. The techniques for eliminating duplicity in searches and other type of raw data used for this review are available to interested individuals at the Zenodo repository at the following address: https://doi.org/10.5281/zenodo.6310254.

## Supporting information

S1 Checklist (DOCX)

S1 Table (DOCX)

S2 Table (DOCX)

S3 Table (DOCX)

S4 Table (DOCX)
