# Supplementary material for: Beyond depression and anxiety; a systematic review about the role of corticotropin-releasing hormone antagonists in diseases of the pelvic and abdominal organs
Source: PLoS One. 2022 Mar 11;17(3):e0264909. doi: 10.1371/journal.pone.0264909 (PMC8916623; doi:10.1371/journal.pone.0264909)
Supplement: S1 Table — (DOCX) [file pone.0264909.s002.docx]

S1 Table: Supporting information for Table 1

| **Study authors** | **Year** | **Animal bodyweight (g)** | **Drug** | **Concentration** |
| --- | --- | --- | --- | --- |
| Ataka K et al. | 2007 | 200-250 | Astressin | 2.5 nmol |
|  |  |  | Antisavagine-30 | 2.5 nmol |
|  |  |  | NBI-27914 | 100 nmol |
| Gourcerol G et al. | 2011 | 280-300 | Astressin 2B | 50 μg/kg |
| Million M et al. | 2013 | 280-320 | NGD 98-2 or NGD 9002 | ﻿30 mg/kg |
| Teitelbaum A et al. | 2008 | 200-250 | Antisauvagine | 50 µg/kg |
| Tsukamoto K et al. | 2006 | 280-330 | Astressin | ﻿10 µg |
| Zhao YX et al. | 2021 | 210-260 | CP 154,526 | ﻿100 mg/kg |
|  |  |  | Astressin 2B | ﻿60 mg/kg |
