# Supplementary material for: Beyond depression and anxiety; a systematic review about the role of corticotropin-releasing hormone antagonists in diseases of the pelvic and abdominal organs
Source: PLoS One. 2022 Mar 11;17(3):e0264909. doi: 10.1371/journal.pone.0264909 (PMC8916623; doi:10.1371/journal.pone.0264909)
Supplement: S2 Table — (DOCX) [file pone.0264909.s003.docx]

S2 Table: Supporting information for Table 2

| **Study authors** | **Year** | **Body weight (g)** | **Stressor** | **Duration of stressor** | **Drug** | **Concentration** |
| --- | --- | --- | --- | --- | --- | --- |
| Yoshimoto S et al. | 2011 | 250-300 | Water avoidance, forced swim, cold restrain and restrain | Homotypic: 5 days, same stressor; Heterotypic, 7 days two different stressors per day. | Astressin 2B | 10µg |
|  |  |  |  |  | NBI-27914 | 100µg |
| Barreau F et al. | 2007 | - | Neonatal maternal deprivation | Three hours a day on postnatal days (PND) 2-14 | ⍺-helical CRF 9-41 | ﻿250 μg/kg |
|  |  |  |  |  | SSR-125543 | ﻿10 mg/kg |
| Li B et al. | 2017 | - | Neonatal maternal separation | Three hours a day on PND 5-9 | Astressin | ﻿60 μg/kg/day |
|  |  |  |  |  | Astressin 2B | ﻿150 μg/kg/day |
|  |  |  |  |  | Antalarmin | ﻿20 mg/kg/day |
| Van den Wijnguard RM et al. | 2012 | - | Neonatal maternal separation | Three hours a day on PND 2-14 | ⍺-helical CRF 9-41 | ﻿250 μg/kg |
| Boucher W et al. | 2009 | - | Acute restrain stress | Single session of 30 min. | Antalarmin | ﻿10 μM |
|  |  |  |  |  | Astressin 2B | ﻿10 μ M |
| Bulbul M et al. | 2019 | ﻿250–300 | Acute restrain stress | Single session 90 min. | Astressin | ﻿100 µg/kg |
| Gourcerol G et al. | 2009 | ﻿20–25 | Acute and repeated restrain stress | Acute: single session -60 min. Repeated: 14 days, 60 min sessions. | Astressin B | ﻿100 µg/kg |
| Kim D et al. | 2010 | ﻿250–300 | Acute restrain stress | Single session of 30 min. | Astressin | ﻿20 μg/kg |
| Nakade Y et al. | 2007 | ﻿250–300 | Acute restrain stress | Single session of 90 min. | Astressin | 10 μg |
| Taguchi R et al. | 2017 | 179-207 | Wrap restrain stress | Single session 20 min. | E2508 | ﻿10, 30 and 100 mg/kg |
| Bradesi S et al. | 2008 | 250-275 | Water avoidance stress | One hour for 10 days. | SSR149415 | ﻿0.3, 1, or 3 mg/kg |
|  |  |  |  |  | DMP969 | ﻿30 mg/kg |
| Buckley M et al. | 2014 | 200-250 | Open field and colorectal distention | Open field: Single session 10 min on experimental day 10. Colorectal distention: Single session 8 min on experimental day 14 | Antalarmin | ﻿10 mg kg−1 |
| Funatsu T et al. | 2007 | - | Conditioned fear (footshock) | 15 trials followed by 30 min re-exposure. | ⍺-helical CRF 9-41 | ﻿0.1 μg |
| Robbins M et al. | 2008 | - | Chronic intermitent footshock | 30 shocks during 7 days | ASVG30 | ﻿12 μg |
|  |  |  |  |  | Antalarmin | ﻿24 μg |
| Seki M et al. | 2019 | ﻿179–203 | Psychological stress: footshock to one rat while the other observed and listened. | 120 min daily for 7 days. | Antalarmin | ﻿200 μg/kg |
| Itomi Y et al. | 2020 | ﻿250-370 | Fear conditioning | 15 trials followed by 15 min re-exposure | T-3047928 | ﻿1-10 mg/kg |
| Roemer E et al. | 2016 | - | Acute stressors: Brief handling, novel environment and partial restrain | Handling: 2 min; other two stressors, 60 min each. | Astressin B | ﻿100 μg/kg |
