# Supplementary material for: Beyond depression and anxiety; a systematic review about the role of corticotropin-releasing hormone antagonists in diseases of the pelvic and abdominal organs
Source: PLoS One. 2022 Mar 11;17(3):e0264909. doi: 10.1371/journal.pone.0264909 (PMC8916623; doi:10.1371/journal.pone.0264909)
Supplement: S3 Table — (DOCX) [file pone.0264909.s004.docx]

S3 Table: Supporting information for Table 3

| **Study authors** | **Year** | **Bodyweight (g)** | **Drug** | **Concentration** |
| --- | --- | --- | --- | --- |
| Im E et al. | 2011 | - | Astressin 2B | 30 μg/kg |
|  |  |  | Antalarmin | 20 mg/kg |
| Gong SS et al. | 2018 | - | Astressin 2B | ﻿20 µg/kg |
| Hoffman JM et al. | 2016 | - | Astressin 2B | 30 μg/kg |
| Jia F et al. | 2013 | - | ⍺-helical CRF 9-41 | ﻿50 µg |
|  |  |  | NBI-27914 | ﻿50 µg |
| La JH et al. | 2008 | 270-310 | Astressin | ﻿30 µg/kg |
| Kokkotou E et al. | 2006 |  | Astressin 2B | 3, 30 or 300 ﻿µg/kg |
| Kubo Y et al. | 2010 | 240-280 | NBI-27914 | ﻿10 mg/kg |
|  |  |  | Astressin 2B | ﻿60 µg/kg |
|  |  |  | Astressin | ﻿50 µg/kg |
| Larauche M et al. | 2009 | 200-250 | Astressin B | ﻿100 µg/kg |
|  |  |  | CP154,526 | ﻿20 mg/kg |
| Liu L et al. | 2011 | - | Antalarmin | ﻿10 mg/kg |
| Nakade Y et al. | 2007 | 250-300 | Astressin | ﻿10 µg |
| Nozu T et al. | 2014 | 200-250 | Astressin 2B | ﻿200 μg/kg |
| Nozu T et al. | 2019 | 300 | Astressin 2B | ﻿100 μg/kg |
| Saito Nakaya K et al. | 2008 | 180-210 | CP154,526 | ﻿20 mg/kg |
