# Supplementary material for: Beyond depression and anxiety; a systematic review about the role of corticotropin-releasing hormone antagonists in diseases of the pelvic and abdominal organs
Source: PLoS One. 2022 Mar 11;17(3):e0264909. doi: 10.1371/journal.pone.0264909 (PMC8916623; doi:10.1371/journal.pone.0264909)
Supplement: S4 Table — (DOCX) [file pone.0264909.s005.docx]

S4 Table: Supporting information for Table 4

| **Study authors** | **Year** | **Bodyweight (g)** | **Drug** | **Concentration** |
| --- | --- | --- | --- | --- |
| Grandi D et al. | 2008 | 180-200 | ⍺-helical CRF 9-41 | ﻿25 µg/rat |
| Torres Reveron A et al. | 2018 | 190-220 | Antalarmin | 20 mg/kg |
| Takeuchi K et al. | 2016 | 200-260 | NBI 27914 | ﻿10 mg/kg |
|  |  |  | Astressin | ﻿3 – 10 µg/kg |
|  |  |  | Astressin 2B | ﻿60 µg/kg |
| Wood SK et al. | 2013 | 300 | NBI 30775 | ﻿10 mg/kg |
